# Ionospheric Narrowband and Wideband HF Soundings for Communications Purposes: A Review

**DOI:** 10.3390/s20092486

**Published:** 2020-04-28

**Authors:** Marcos Hervás, Pau Bergadà, Rosa Ma Alsina-Pagès

**Affiliations:** 1 GTM—Grup de recerca en Tecnologies Mèdia, La Salle—Universitat Ramon Llull, c/Quatre Camins, 30, 08022 Barcelona, Spain; 2 Wavecontrol, c/Pallars, 65-71, 08018 Barcelona, Spain

**Keywords:** HF, vertical sounding, oblique sounding, skywave, ionosphere, communication, Doppler spread, delay spread, SNR

## Abstract

High Frequency (HF) communications through ionospheric reflection is a widely used technique specifically for maritime, aeronautical, and emergency services communication with remote areas due to economic and management reasons, and also as backup system. Although long distance radio links can be established beyond line-of-sight, the availability, the usable frequencies and the capacity of the channel depends on the state of the ionosphere. The main factors that affect the ionosphere are day-night, season, sunspot number, polar aurora and earth magnetic field. These effects impair the transmitted wave, which suffers attenuation, time and frequency dispersion. In order to increase the knowledge of this channel, the ionosphere has been sounded by means of narrowband and wideband waveforms by the research community all over the world in several research initiatives. This work intends to be a review of remarkable projects for vertical sounding with a world wide network and for oblique sounding for high latitude, mid latitude, and trans-equatorial latitude.

## 1. Ionospheric Historical Context

The first steps in wireless communications led us back to the Treatise on Electricity and Magnetism [1], by J.C. Maxwell, presented in 1873. It somehow founded the current modern electromagnetism propagation theories; nevertheless, it was not until 1888 when the first radiowaves were effectively detected by H.R. Hertz, who demonstrated Maxwell’s theory by means of the perturbation over a spark coil. This work later inspired G. Marconi, who started experimenting with wireless telegraphy; he sent messages in Morse code using a radiolink in 1896. It was not only until 1901, when a 3000-km transatlantic telegraphic signal was detected, breaking the theory that any radiolink required a direct line-of-sight (LOS) to propagate [2]. It was in the 1920s when E. Appleton [3] managed to demonstrate that signals could be detected without LOS anywhere in the planet by means of the reflection over an electrically-charged region of the atmosphere [4]. It was around 1950 when the Institute of Radio Engineers (IRE) defined the ionosphere as the *part of the outer atmosphere where ions and electrons are present in quantities sufficient to affect the propagation of radio waves* [5]. This region of the Earth’s outer atmosphere begins around 60 km high and includes the thermosphere, as well as parts of the mesosphere and the exosphere [6,7].

**Figure 1:**
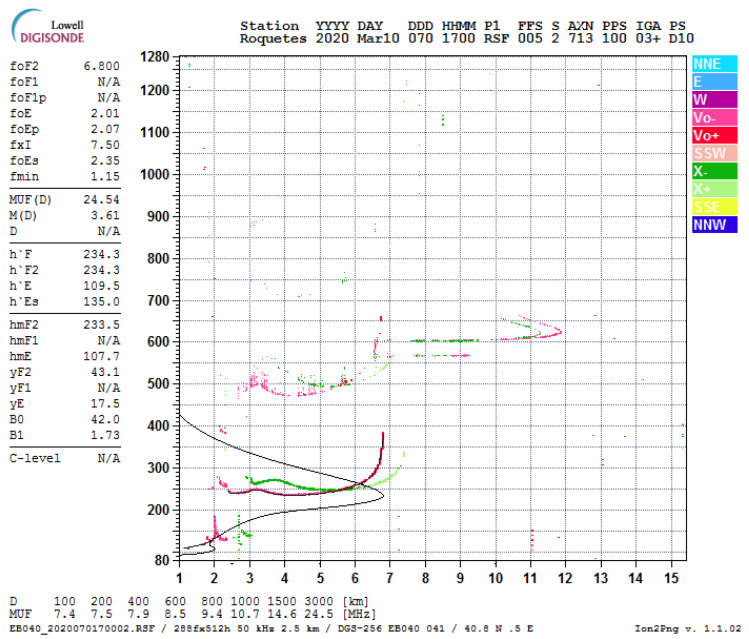
Ionogram of 10 March 2020 at 17 UTC recorded in Roquetes Station (Spain) offered by the Ebre Observatory. Available online at http://obsebre.es/en/checked-ionograms (accessed on 14 March 2020).

Communication researches have been widely interested in the study of the ionosphere, due to the fact that it is a time and frequency dispersive channel in the HF range [8]. The approach to the characterization of the ionospheric performance as a communication channel is mainly conducted by means of a ionosonde, which is a transmitter that covers the entire HF frequency range with an antenna presenting an homogeneous efficiency in all the frequency range, and it is working with a vertical setup. The echoes of the transmitted signals are received, analyzed and displayed in a representation called *ionogram*, which shows the reflection altitude as a function of the emitted frequency, as observed in the example of ionogram in Figure 1. The black line corresponds to the computed plasma frequency in relation to the true altitude, and the ordinary frequencies following the right hand circular polarization are represented in red, while the extraordinary frequencies that follow the left hand circular polarization are shown in green (assuming upward wave propagation and a northern-hemisphere location [8]).

Nevertheless, the communication characteristics nowadays require further methods to evaluate the quality of the communications, including not only narrowband tests but also wideband and other channel parameters required for the design of the HF system. In order to predict the channel performance, parameters as the best usable frequency, the expected signal-to-noise ratio (SNR) and the elevation angle, and of course, in some situations, the multipath spread estimation are crucial values to take into account when designing an HF ionospherical communication system. In this sense, it is worth mentioning IONCAP [9] and its variations VOACAP and ICEPAC (“Voacap quick guide”, www.voacap.com, last access 30/10/2018.), and also ASAPS [10]. All these simulators use virtual geometry (flat Earth models, laminar ionosphere, etc.) to minimize the computation time, at the cost of certain lack of accuracy in the outcome of the simulation depending on the latitude. These techniques are frequently used when the communication link under test is not yet available, or in the first stages of the design of the communication link. The most realistic approach to a channel performance prediction is to sound the link widely, both narrowband and wideband, at all available frequencies, hours of the day and time of the year throughput a whole solar cycle.

This article pretends to review a set of relevant projects focused on designing and testing narrowband and wideband, both vertical and oblique, sounding techniques. In Section 2, the theory of the characteristics of the ionosphere and its variability and dependencies has been reviewed. In Section 3, several case studies have been analyzed. Finally, the concluding remarks are covered in Section 4.

## 2. Ionosphere Characteristics

In this section we detail the physical characteristics of the ionosphere that influence the propagation of the electromagnetic signal, and the analyzes that, from a communications point of view, can be performed to expand the knowledge of a specific ionospheric channel for the propagation of telecommunication signals.

### 2.1. The Regions of the Ionosphere in Terms of Latitude

The Earth is divided into three regions regarding the ionosphere, which present different characteristics due to their geomagnetic latitude [11]. The mid-latitude is the widest studied and described. The ionization is produced almost entirely by energetic ultra-violet and X-ray emissions from the Sun. These processes are also present in high-latitude and low-latitude, but other factors produce major changes to the performance of the ionosphere. For instance, absorption of radio signal strength varies depending on the latitude. For mid-latitude and equatorial regions, radiowaves of frequency above 70 MHz will assure penetration of the ionosphere without significant absorption; whereas, at 30 MHz the absorption of one-way vertical incidence signals can have typical values of 0.2 to 0.5 dB, which can go up to 5 dB during solar flares [12].

Low latitude regions, around ${\left[20,30\right]}^{\circ }$ around both sides of the magnetic Equator, are very influenced by electromagnetic forces that appear because geomagnetic fields run horizontally over the magnetic equator [11]. The electrical conductivity is larger than usual over the Equator, forming a current named *electrojet* and being subject to electrodynamic lifting and *fountain effect*, distorting the general form of the ionosphere. Scintillation on Earth-space paths, which is a rapid fluctuation of radio-frequency signal phase and/or amplitude, generated as a signal passes through the ionosphere, is quite severe in low latitudes as well as in high latitudes, with observed effects up to gigahertz frequencies [12]. In equatorial latitudes the scintillation peak activity occurs at vernal, as well as autumnal equinox, mostly after the ionospheric sunset and events can last from 30 min to hours.

Finally, in high latitudes the behaviour is the opposite [11,13]. In this region, the electromagnetic field runs nearly vertically, and this leads to a more complex situation in the ionosphere than in any other of the latitudes [12]. The magnetic field-lines connect the high latitudes to the outer part of the magnetosphere, driven by solar wind. This implies four essential consequences:

- The high-latitude ionosphere is clearly dynamic.
- The Sun energy particles access easily to this region, and produce an additional ionization, most of the times affected also by sporadic events, worsening the polar radio propagation.
- The high-latitude region is also the auroral zone. This phenomena includes also *electrojets* and *substorms*, where the rate of ionization is greately increased due to the arrival of energetic electrons.
- A zone of lesser ionization can be formed between the auroral and the mid-latitude ionosphere, despite the mechanism of formation of the trough are not clear.

These consequences, among others that are explained in Reference [13], cause that any propagation in high-latitudes may be affected by one or more of those deformations, and have to be studied individually and with detail. Regarding absorption at high latitudes, enhanced levels of absorption can occur due to polar cap and Auroral events, which occur at random intervals, last for different periods of time, and their effects are functions of the locations of the terminals and the elevation angle of the path [12].

### 2.2. Ionosphere Layers: Day-Night and Latitude Dependencies

The ionosphere is the ionized part of the Earth’s atmosphere. It is formed by a group of electrons and electrically-charged particles, starting at an approximate altitude of 60 km. It is a part of the atmosphere with intense activity because it is affected by several natural phenomena, as winter and equatorial anomalies [14,15], and also the equatorial *electrojet* [16]. Another interesting issue is the periodic day-night variance, which exists in the ionosphere due to the main source of ionization, that is, the Sun [8]. It is detailed that during daytime the gases in the atmosphere are ionized by means of the absorption of photons of short wavelength coming from the Sun. In this sense, most of the electron density loss occurs during the night, when electrons disappear due to the influence of gas dynamics and electromagnetic interactions. The phenomena of diffusion and proton absorption provides a path for the removal of the molecular ions [17].

Several travelling ionospheric disturbances, anomalies as sporadic-E, spread-E or spread-F affect the ionosphere specially at certain seasons and latitudes, and it makes its evolution variable between days, seasons and also geographic fluctuations [18]. Furthermore, solar flares and geomagnetic storms are also present in the ionospheric channel and are unpredictable [8]. The sunspot number has a pseudo-periodic cycle of between 9 and 14 years length [19]. Solar radiation is the main influence on ionization of the heterogeneous plasma in the ionosphere. It mainly depends on the solar flux and the position and angle of the Sun in relation to the Earth’s ionosphere. Therefore the ionosphere layers definition is highly correlated with the day-night cycle.

The D layer (found in ∼60–80 km) contains free electrons that recombine with oxygen ions during the night, forming uncharged oxygen molecules. During daytime, the D layer attenuates low-frequency radio waves of the HF band and during the night disappears [20]. The E layer (found between ∼80–160 km) does not disappear at night despite its low ionization. Ionization in this layer appears mainly for soft X-ray and residual nighttime ionization is due to cosmic rays among other sources. This residual ionization is usually unimportant for HF links except in high latitudes, where it varies rapidly due to energetic particles precipitating [21,22].

The $E_{s}$ layer (found between ∼80–120 km), also known as sporadic E, is described as thin layers of intense ionization, that appear rarely and for a short period of time [23]. It is more frequent in summer and during the day [8]. Finally, the F layer (found between ∼160–400 km), comprises two peaks of ionization during daytime and only one layer during nighttime. It is the layer with the higher density of free electrons and its ion distribution varies from day to night. During the day, it contains a small peak of ionization called F1 and the dominant F2 is also observed, carrying the main electron density. The F layer and the E layer are the only ones that remain during the 24-h cycle. Although the F layer contains the main ionization peak, which is dependent on available frequencies, link length and ionization level, the E layer also plays an important role in the possibilities of electromagnetic signal transmission.

### 2.3. Ordinary and X-Ordinary Waves: Description and Latitude Dependencies

The ionosphere consists of plasma ionized by the solar activity superposed with the Earth’s magnetic field. These two factors make it an anisotropic medium. When an incident radio wave impacts the ionosphere, it splits into ordinary and extraordinary components due to the influence of the magnetic field [8]. These modes are differently affected by the external magnetic field. In this sense, the index of refraction in the ionosphere is defined by the Appleton formula [24], which provides different results for both ordinary and extraordinary waves. In fact, according to this formula, only these two modes can propagate in this medium.

The ordinary and extraordinary modes propagate on different paths and velocity due to their distinct refractive index. As a consequence, these waves are reflected back at different heights reaching any receiver with different delays. Moreover, the amplitude and the maximum reflected frequency are also different. In fact, the maximum reflected frequency in the F2 layer for the ordinary (fof2) is usually lower than that for the extraordinary (fxf2). Ordinary and extraordinary waves have elliptical polarization with an axial ratio that might be different.

In the particular case of high-latitudes [25], where the magnetic field is almost vertical, the extraordinary waves have horizontal polarization for near grazing incidence. As the extraordinary mode waves are heavily absorbed, a vertical HF antenna, that would receive the ordinary mode should take advantage over a horizontal antenna. Nevertheless, most of the times dipoles are used in that situation for convenience.

### 2.4. Technical Procedures and Metrics to Analyze the Ionospheric Variations

The ionosphere is a dynamic medium in terms of propagation, especially if working with low-latitude or high-latitude. In this sense, in the procedure of design of a communications system to work exhaustively in a ionospheric environment, there are several preparatory processes to be conducted, and a set of metrics to analyze depending on the frequency of transmission, the latitude and the hour of the day or night.

A sounding of the channel should be done, with the wider possible set of requirements to detect the best possible setup for the particular link. For this purpose, several metrics are defined to support the optimum setup depending on the sounding results.

#### 2.4.1. Oblique and Vertical Sounding

In telecommunication systems, a ionospheric sounding is a technique that can provide real-time data about radio propagation conditions over an HF link, by means of a basic system consisting on having a synchronized transmitter and receiver [22]. In this sense, the time delay between the transmission and the reception can be translated to the identification of the effective ionospheric layer altitude.

Vertical incidence sounding normally uses a collocated transmitter and receiver and involves a certain number of preselected frequencies, which are directed vertically to the ionosphere. The values of the reflected returned signals are used to determine the ionospheric layer altitude in an effective term [26]; this is also the technique used to determine the critical frequency. Oblique incidence sounding uses a transmitter in one end and a receiver on the opposite; the distance between those two devices is the effective channel to be evaluated (in terms of ionosphere). The receiver has to be synchronized to the transmitter, and usually deploys a ionogram as a result (see Figure 1). The transmitter uses either a stepped frequency signal or a swept frequency signal over the ionospheric channel, which is measured at the receiver. The ionogram, as depicted in Figure 1, pretends to display the real altitude as a function of frequency by means of the virtual altitude measurement using the time delay.

#### 2.4.2. Narrowband and Wideband Techniques: SNR, Availability and Time and Frequency Dispersion

Narrowband sounding is commonly used to monitor the channel and gather information on the reliability of HF communications. For instance, the reception of WWV Standard Frequency transmissions can be used to estimate the range of frequencies with good availability, to study its relationship with the sunspot number and even characterize the channel during disturbed periods when the ionosphere is under the effect of a geomagnetic storm. When assessing the effect of a disturbance on a communication system it is not sufficient to consider only signal strength. The nature of the signal must also be considered and hence the depth and rate of fading and the time and frequency spreading of the signal must be studied. Wideband sounding is then used to describe these distortion effects that suffer the transmitted signals on a given bandwidth.

There are many techniques to sound the channel and to collect the required propagation parameters needed to design a communication link. Reference [27] revises several techniques to sound a radio channel (i.e., single-tone continuous wave, spaced tone waveform, pulse waveform, pulse compression waveform and coded pulse signals). All of them use different kind of waveforms designed to detect the prevailing propagation conditions. Furthermore, several architectures and methodology have been proposed in the literature [28,29,30] to obtain the key parameters of the ionospheric channel, such as the following: (i) Frequency and temporal dispersion by means of the scattering function, (ii) Time of flight, (iii) Signal to noise ratio, (iv) Link power attenuation, (v) Phase stability, (vi) Angle of arrival and (vii) Independent measurements for ordinary and extraordinary rays.

## 3. State of the Art of Sky Wave Sounding

Signals can be transmitted beyond line-of-sight (BLOS) in HF communications through different mechanisms: (a) ground wave, (b) single sky-wave, (c) multi-hop skywave, (d) sporadic-E, (e) and so forth. These propagation modes and other ionospheric effects lead to the degradation of the signal, which suffers attenuation and time and frequency dispersion (Doppler spread and Doppler shift).

In this section we review several projects whose shared goal has been the study of some or all of these impairments with the aim to improve HF communications and satellite navigation. First of all, we review a Radiocommunications section of the International Telecommunications Union (ITU-R) report on recommendations on HF field-strength measurement campaigns, we then go through a project on vertical ionospheric sounding and we finally address oblique sounding by means of three different projects focused on three different latitudes, that is, high, middle and trans-equatorial latitudes.

### 3.1. Recommendation ITU-R P.845-3

The Radiocommunications section of the International Telecommunications Union (ITU-R) considered in 1997 that the HF field-strength prediction methods should be compared against measured field- strength data with a certain level of accuracy [31]. It also considered that accurate field-strength data should be indispensable for the use of HF spectrum. Therefore, it published some recommendations on:

- Measurement of sky-wave signal intensities above 1.6 MHz;
- HF field-strength measurement technique. Specifications for field-strength measurement campaign with the aim to improve future prediction methods.

#### 3.1.1. Measurement of Sky-Wave Signal Intensities above 1.6 MHz

ITU, as a standardization organization, recommends that the measurements of sky-wave signal intensities should be undertaken in a carefully controlled manner to help improve the accuracy of methods for estimating field-strength and transmission loss. Ideally, measurements should be carried out systematically over as wide a range of conditions as possible at a series of frequencies over paths of different lengths in all regions of the world. Measurements should be done at each hour of the day in the separate seasons and for a whole solar period.

ITU recommends that, for the sake of interpretation and being statistically meaningful, measurements should cover a minimum period of one year at a given fixed frequency. ITU also exposes the advantages of recording signals simultaneously over a path at a series of different frequencies, both to aid the understanding of propagation effects and to permit quantitative data to be obtained by night when maximum usable frequencies are low, as well as by day when there is much absorption at the lower frequencies and signals might be masked by background noise.

##### Transmitter

The transmitter should preferably operate 24 h per day and should transmit an unambiguously identifiable signal to prevent mistaking the signal of interest for co-channel signals, adjacent channel signals or interfering noise. The transmitted signal should be stable both in frequency and radiated power, which should be higher than 1 kW for short paths and higher than 10 kW for medium and long paths. If the transmitter uses modulated signals the type of modulation should be constant and the mean percentage modulation should not vary. ITU suggests to better use narrow-band transmissions (i.e., 1 kHz or less) rather than wide-band transmissions to reduce the probability of being interfered.

According to ITU recommendations non-directive antennas have advantages over directive antennas because the relative strength of the received signals coming from different paths are mainly determined by propagation effects and not for the radiation pattern. Another benefit of omnidirectional antennas is the fact that in absence of knowledge of wave launch directions, valid deductions can only be made in case of a single transmitter-antenna gain. Vertical half-wave dipoles with all-round azimuthal coverage are preferred for monitoring and sounding purposes since radiation patterns may be fairly estimated, even at low elevation angles.

##### Receiver

Prediction methods of signal strength do not take into account local characteristics at the receiver site and consequently the receiver antenna should be clear of obstacles (e.g., foliage and buildings) and adjacent antennas should be separated more than ten times the antenna length. Due to the high level of noise and interference at the HF band, ITU recommends not to use high gain receiving antennas. Therefore, appropriate to employ should be a short vertical antenna or a grounded vertical monopole shorter than a quarter wavelength or a small loop antenna. In case of using a loop antenna it should be aligned in a vertical plane containing the great-circle path to the transmitter and in case of long-distance paths, where off-great circle propagation contribution is important, vertical monopoles are recommended due to its omnidirectional azimuthal radiation diagram. Receiver setup should also take into account other issues such as the ground slope, the radiation pattern elevation angle and the ground conductivity.

Appropriate impedance matching should be provided between antenna and transformer or wideband pre-amplifier. In order to maximize signal-to-noise relation the receiver bandwidth should be consistent to the bandwidth of the transmitted signals.

#### 3.1.2. HF Field-Strength Measurement Technique

In order to minimize uncertainties that may impair an HF field-strength measure campaign and to provide high quality data ITU suggests a number of arrangements in both transmitter and receiver setups.

The transmitters are recommended to be frequency agile, radiating in up to five frequency bands, preferably in fixed frequency bands (e.g., 5.5, 8, 11, 15 and 20 MHz), sequentially with steady signal periods combined with coded sequences to allow computer identification of the source as well as computer evaluation of the intensities of both signal of interest and background noise or interference. With the goal to gather data representative of HF propagation characteristics ITU proposes to locate at least nine transmitters spread over the three world regions (i.e., Europe/Africa, America and Asia/Australasia) on both Hemispheres, at middle latitudes and in the tropics. Broadband antennas, omnidirectional in azimuth, with broad elevation pattern, such as broadband monopoles or conical structures, are recommended. A single antenna could be used to cover the whole HF frequency range (i.e, from 2 to 30 MHz) to avoid switching high RF powers (i.e., between 1 and 5 kW) along with a frequency selective matching circuit to operate on the lowest operating frequency band.

Transmitted signal should be of type F1B (i.e., FSK modulated telegraph signal) modulated with Frequency Shift Keying (FSK) with a frequency shift of 850 Hz. The transmitted sequence should have a duration of 12 s, be repeated for 4 min and be composed by the following parts:

- FSK preamble at 100 bit/s for 1 s, comprising signal reversals commencing on the mark frequency;
- A pause of 50 ms;
- An identification signal in Morse code on the higher frequency contained within a period not exceeding 3.3 s;
- A pause of 50 ms;
- A 256-bit complementary sequence transmitted at 1200 bit/s as follows: Sequence 1 = 111001000010100000010100110110000001101100101000111010111101100000011011110101 1100010100110110001110010011010111111010 11110110000001101111010111111010110010011 1000110110010100011101011110110001110010000101000111010110010011 111100100110101111110101111011000;
- A pause of 50 ms;
- A seconds 256-bit complementary sequence transmitted at 1200 bit/s as follows: Sequence 2 = 0001101111010111111010110010011111100100110101110001010000100111000110 1111010111000101001101100011100100110101111110101111011000111001000010 1000000101001101100011100100110101110001010000100111111001000010100011 1010110010011111100100110101111110101111011000;
- A pause of 50 ms;
- A series of FSK reversals, comprising 273 bits at 100 bit/s, commencing on the lower frequency;
- A 127 bit Gold code identifier sequence [32], at 100 bit/s, commencing on the lower frequency;
- a constant signal on the higher frequency for a duration of at least 3 s, which should continue until the total time for the sequence is 12 s.

To receive the signals coming from the nine proposed transmitters there should be as many receivers as possible placed not nearer than 500 km to any transmitter due to the null in the elevation pattern of the omnidirectional transmitting antenna. Short vertical active antennas placed on flat ground within an area of homogeneous and good ground conductivity, with no obstacles within 25 m radius and away from any metallic structure are recommended. The field-strength receiver should comply with the following minimum performance requirements:

- synthesizer control (10 Hz step);
- external bus available for computer control;
- frequency accuracy ±1 part in $10^{6}$;
- synthesizer noise sidebands: reciprocal mixing performance better than 70 dB in a bandwidth of 3 kHz at 20 kHz offset;
- sensitivity SSB 1.0 mV terminated for at least 10 dB (S + N) /N for a bandwidth of 3 kHz;
- unwanted spurious responses (e.g., image, IF) better than 70 dB;
- selectivity: approximately 3 kHz bandwidth, shape factor (−60 dB to −6 dB) 2:1;
- linearity: 3rd-order intercept point, 20 kHz spacing, +10 dBm;
- true average measuring capability within 4 s;
- timing accuracy within the receiver system should be maintained within 1 s.

For each combination of transmitter, receiver and frequency the receiver should gather sufficient samples within each hour to allow statistically meaningful estimates of the hourly medians of both the signal and noise intensities, which is determined in relation to the likely within-an-hour fading. Using either analogue or digital signal processing, average voltage amplitudes over a period of 4 s within each 12 s transmission cycle should first be determined. A minimum of 12 such samples within a given hour on a given day should be taken, uniformly distributed over the transmitting period.

### 3.2. GIRO

**Figure 2:**
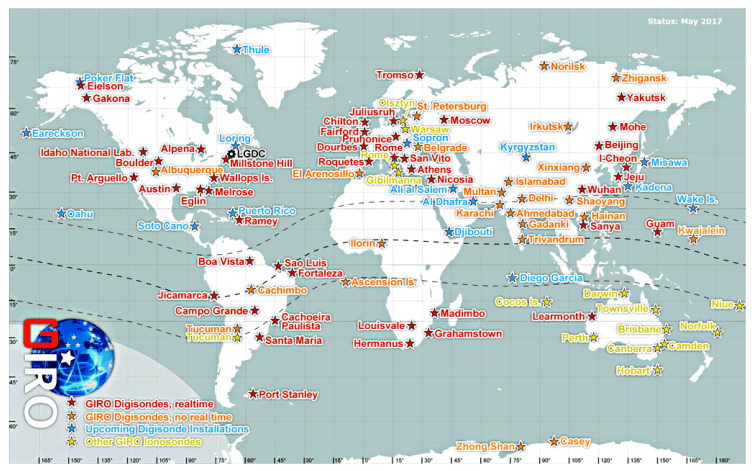
Map of digisondes contributing data to the Global Ionospheric Radio Observatory (GIRO). Red stars identify online digisondes providing data to GIRO in near-real-time, orange stars denote digisondes providing data to GIRO in no real time, yellow stars denote other type of ionosondes that also contribute to GIRO archives, and blue stars correspond to digisonde installations planned for the next future.

Global Ionospheric Radio Observatory (GIRO) is a project that provides access to a global network of digital ionosondes (*digisonde*), which most of them fed the GIRO database in real time. GIRO consists of three main parts: (i) a network of 64 digisonde stations providing online (42 out of 64) and offline data (see map on Figure 2), (ii) two databases, the Digital Ionogram Data Base (DIDBase) and the DriftBase for skymap/drift measurements and (iii) an associated software capable of automatic and interactive data and the derivation of higher order products for end user applications [33].

**Figure 3:**
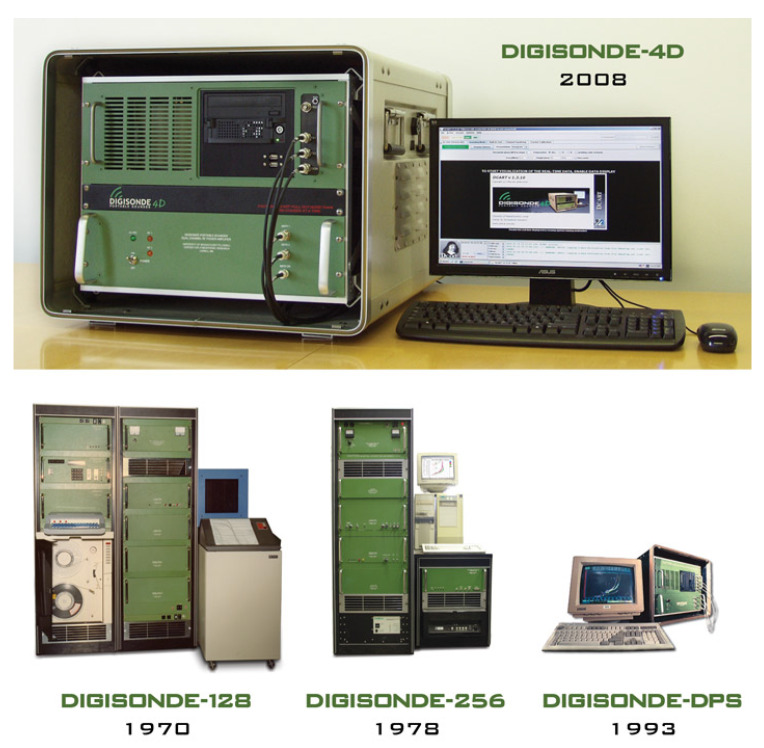
Evolution of digisonde sounders since 1969.

A digisonde is a radar that uses high-frequency waves for remote sensing of the ionosphere. Lowel Digisonde International develops and manufactures these equipment in collaboration with UMass Lowell Center for Atmospheric Research. Since 1969 they have developed up to 4 different versions of this equipment (see picture in Figure 3) and the current version is the DPS4D, which monitors the effects of space weather on Earth’s ionosphere, supports communication and navigation satellite operations as well as HF and VHF radiowave communications. Its main technical characteristics can be summarized as follows.

Regarding the transmitter device:

- Frequency scan range from 0.5 up to 30 MHz with a size step selectable to 1 kHz;
- Ionogram scan time from 2 up to 200 s;
- Fully digital frequency synthesis (frequency switching time <1 μs);
- Pulse repetition rate of 100 and 200 pps;
- 533 μs (16 chips of 33 μs) waveform with 30 kHz signal bandwidth;
- 2 transmit antenna connectors for turnstile transmit antennas;
- Dual RF MOSFET amplifiers for polarized transmission;
- A peak pulse power at each of the channels of 150 W.

Regarding the receiver device:

- Frequency range from 0.5 up to 30 MHz;
- Bandwidth of 34 kHz;
- Noise figure of 11 dB at the receiver preamplifier;
- Receiver sensitivity down to −130 dBm;
- Instantaneous dynamic range of 90 dB;
- 4 receive antenna connectors for crossed magnetic dipole antennas

Regarding the signal processing capacity:

- Two Embedded Intel Core 2 Duo Dual Core processor SBCs;
- Selectable range of bins (256 or 512);
- Height range from 0 up to 1200 km;
- Height resolution of 2.5 km (500 m sample spacing if using differential phase technique);
- Processing pulse compression waveform of 16 chips provides 15 dB of processing gain;
- Integration of 4 to 128 waveform provides a processing gain up to 21 dB;
- Doppler range of ±3 Hz to ±50 Hz;
- Doppler resolution of 0.0125 Hz to 12.5 Hz;
- Amplitude resolution of <0.01 dB;
- Alternating transmission with O and X, synchronized receive antenna polarizations.

Among the records provided by a digisonde, the ionogram is the most classical. The system performs a frequency sweep and provides a representation of the virtual height (according to time of flight) as a function of frequency. The virtual height is greater than the real height since the ionosphere behaves as a dispersive medium and radio pulses travel slower than light. However, by applying inversion ionogram algorithms it is possible to compute a real height profile of the electronic density. Besides, the Total Electron Content (TEC) could be also estimated through the integration of the electron density profile.

The skymap is another type of record provided by a digisonde. It gathers, for both a certain band of frequencies and a certain rank of reflection heights, the incoming angle of reflected pulses in the ionosphere (azimuth and elevation), and their Doppler shift. This information is processed to derive the place on the vertical axis where the radio pulses have been reflected and it is able to show if the reflective layer is tilted or if it presents irregularities. Moreover, it can also show if the reflection points move respect to the transmitting source (if the reflective layer is dynamic), providing the apparent drift speed of the reflective layer.

The Lowell GIRO Data Center (LGDC) implements a suite of technologies for post-processing, modeling, analysis and dissemination of the acquired and derived data products such as:

- IRI-based Real-time Assimilative Model (IRTAM), that builds and publishes every 15-min an updated “global weather” map of the peak density and height in the ionosphere, as well as a map of deviations from the classic IRI climate;
- Global Assimilative Model of Bottomside Ionosphere Timelines (GAMBIT) Database and Explorer holding 15 years worth of IRTAM computed maps at 15 min cadence;.
- More than 17 million ionograms and matching ionogram-derived records of URSI-standard ionospheric characteristics and vertical profiles of electron density;
- More than 10 million records of the Doppler Skymaps showing spatial distributions over the GIRO locations and plasma drifts;
- Data and software for Traveling Ionospheric Disturbance (TID) diagnostics;
- HR2006 ray tracing software mated to the “realistic” IRTAM ionosphere.

In cooperation with the URSI Ionosonde Network Advisory Group (INAG), the LGDC promotes cooperative agreements with the ionosonde observatories of the world to accept and process real-time data of HF radio monitoring of the ionosphere, and to promote a variety of investigations that benefit from the global-scale, prompt, detailed, and accurate descriptions of the ionospheric variability.

GIRO provides access to Automatic Real-Time Ionogram Scaler with True height (ARTIST) software, which is an “autoscaler” of ionogram traces. Use of real-time ARTIST-derived data as input to ionospheric models and associated algorithms (e.g., ray tracing and optimal frequency allocation) started in 1988 when the USAF Weather Agency (AFWA) established a network of 18 digisondes. Since then, other agencies have built similar networks: (i) the Digital Atmospheric Server (DIAS) became operational in 2004 with 6 contributing digisondes located in Europe; and (ii) the Jindalee Operational Radar Network of the Australian government installed 11 digisondes to provide data to their real time ionospheric model. GIRO data have also been used to update the Boeing Plasma Interaction Model (PIM) in support of spacewalk scheduling for the International Space Station.

### 3.3. DAMSON

The aforementioned ionospheric adverse effects take place anywhere in the world; however, multipath dispersion and Doppler spread is more severe at high latitudes. In 1993, there were very little bibliography about the severity and the frequency of occurrence of these effects at high latitudes. Therefore, the DAMSON (Doppler And Multipath SOunding Network) experiment was deployed to make measurements over a number of mid and high latitude paths [34]. The DAMSON project was a collaboration of the UK’s Defence Evaluation Research Agency (DERA), Norway’s Forsvarets Forskningsinstitutt (FFI), Sweden’s Försvarets Forskningsanstalt (FOA) and Canada’s Communications Research Centre (CRC).

#### 3.3.1. Scope and Objectives

The main objective of the DAMSON project was to collect a large database of measurements over many months at high and mid-latitudes to quantify Doppler spreads, Doppler shifts, multipath delay and signal strengths. These database allowed to fulfill other major objectives: (a) Assessment of HF data modem waveforms: this information will be used for the specification of robust modulation scheme on high-latitudes paths, (b) Updating Propagation Prediction codes: the conducted measurements will derive rules that will be incorporated in propagation predictions, especially on polar-cap and auroral links, and (c) Application to HF Simulators: the measurements will be used to specify HF simulator characteristics to test the communication systems and modems, until the beginning of this project simulators were based on the Watterson model [34].

#### 3.3.2. System Deployment

**Figure 4:**
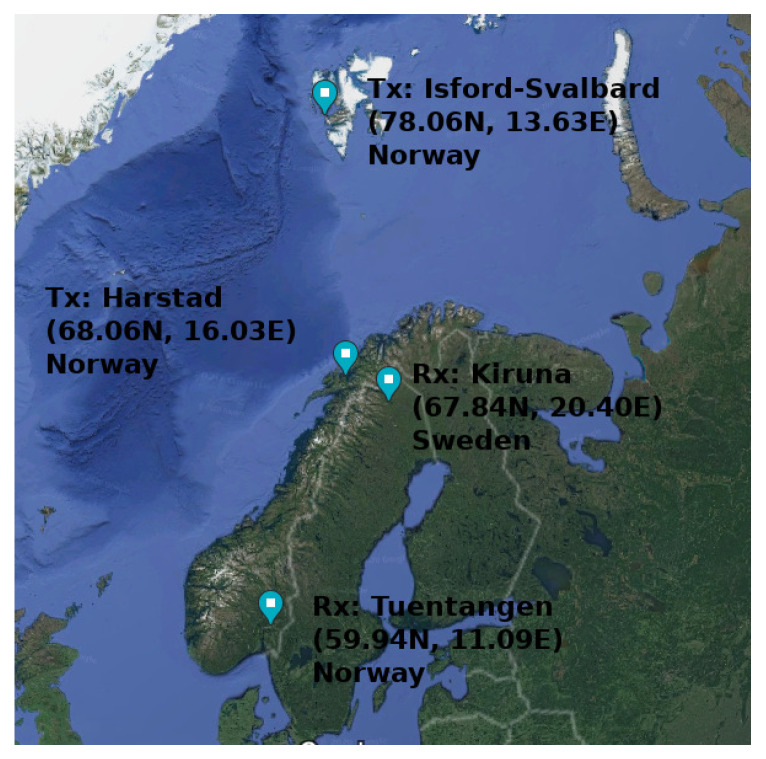
Deployment of the main sites used in the DAMSON project to sound the ionosphere. We can observe auroral, sub-auroral and polar regions.

The project was sounding from mid-1990s to the early 2000s, which is more than half a solar cycle, although it was described around 1994. This system was deployed with two transmitters and two receivers in northern Scandinavia, which are described in Figure 4, with 10 frequencies to characterise all four paths in the auroral, sub-auroral and polar regions. It was sounding every 10 min on each of 10 frequencies from 2.8 MHz to 21.9 MHz for multiple years (24 h/day) in these 4 places, although other sites have also been used for some concrete studies.

The three longest radio links, Isford-Tuentangen, Isford-Kiruna, Harstad-Tuentangen, are approximately north-to-south oriented, and the prediction programs depicted that these links exhibit multi-hop propagation and multi-mode propagation for some frequencies and times-of-day.

- The Harstad-Tuentangen link is placed on a sub-auroral region, except for highly geomagnetically active periods.
- The Isford-Tuentangen link exhibits a multi-hop paths that may reflect from ionospheric regions inside the polar cap, in the auroral zone or below the auroral zone.
- The Isford-Kiruna path, 1150 km, falls almost completely in the auroral region, ensuring that reflection zones are in a highly disturbed region of the ionosphere.

The shortest radio link (180 km), Harstad-Kiruna, is a west-to-east oriented near vertical incidence skywave (NVIS) path. The NVIS maximum range is approximately 200 km, for this reason these links can exhibit ground wave propagation, producing self-interference at the receiver. The DAMSON project has analyzed two NVIS radio links, the aforementioned Harstad-Kiruna and Harstad-Abisko (68.34${}^{\circ }$N 18.83${}^{\circ }$E), which is a 120 km link.

As it was mentioned previously, other radio links were also deployed for particular works. For instance, DAMSON was setup in Thailand in 1997 to measure an equatorial HF channel of a 600 km path. The transmitter was located at 7.2 ${}^{\circ }$N, 100.6 ${}^{\circ }$E and the receiver was located at 12.7 ${}^{\circ }$N, 101.0 ${}^{\circ }$E creating a north-south oriented path.

#### 3.3.3. Measurement Setup

**Figure 5:**
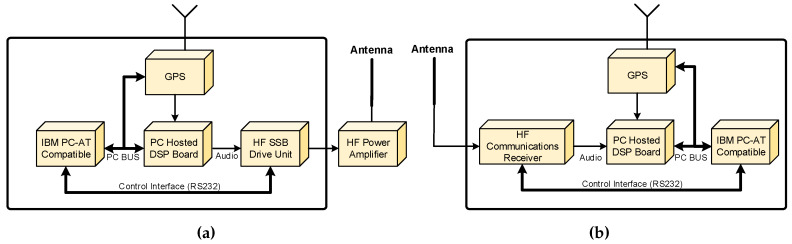
(**a**) Complete block diagram of the DAMSON transmitter and (**b**) Complete block diagram of the DAMSON receiver.

DAMSON setup is an oblique channel sounding system, which has been developed by the UK Defence Research Agency (DRA), made of commercially available equipment such us HF communication receivers and transmitters, computers, and so forth,. and make an extensive use of digital signal processing (DSP) techniques to measure channel parameters using different waveforms [34,35]. This system was designed with a high precision GPS (Global Positioning System) to synchronise in time the receiver and transmitter with an accuracy better than 10 μs, which this synchronizations is mandatory to obtain a measurement of the time of flight of the received signal. A block diagram of the transmitter and receiver setup is depicted in Figure 5a,b.

The main parts and features of this transmitter platform are the following:

- An IBM compatible personal computer (PC) which is used to carry out control functions.
- Configurable parameters such as frequency and transmit bandwidth are controlled by the PC and communicated to other modules using a serial communication interface (RS232).
- A DSP card plugged into the PC to perform both the real time processing and the digital to analogue conversion required to generate waveforms.
- An HF single-sideband (SSB) to modulate the output base band signal of the DSP card. The HF driver has a high stability frequency reference better than 1 ppm and a configurable SSB bandwidth up to 12 kHz.
- The HF output fed a wide band linear Power Amplifier, which feeds the antenna system with a maximum power between 500 and 1000 W.
- A GPS receiver plugged into the PC is used to obtain time synchronization between the transmitter and the receiver.

The main parts and features of this receiver platform are the following:

- An IBM compatible personal computer (PC) which is used to carry out control functions, data storage, data analysis and display.
- Configurable parameters such as frequency and transmit bandwidth are controlled by the PC and communicated to other modules using a serial communication interface (RS232).
- A DSP card plugged into the PC to perform both sampling the received signal with an on board analog to digital converter (ADC), and the real time processing to detect and process received signal.
- An HF single-sideband (SSB) to demodulate the received signal obtaining a base band signal to feed the DSP card. The HF driver has a high stability frequency reference better than 1 ppm. The HF SSB can operate in automatic gain control (AGC) mode or in a manual (controlled by the PC) gain mode.
- A GPS receiver plugged into the PC is used to obtain time synchronization between the transmitter and the receiver.

#### 3.3.4. Measurement Techniques: Waveforms

**Figure 6:**
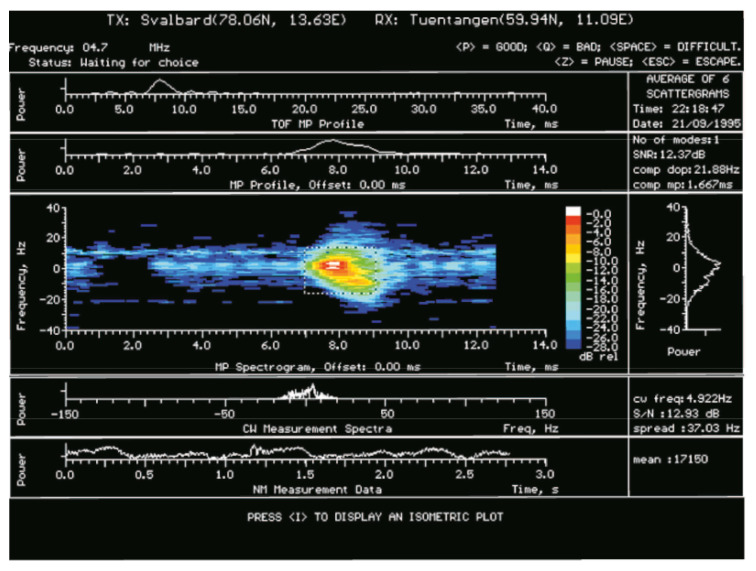
Example DAMSON output, from Reference [36].

There are defined 3 main modes of operation: (a) Delay Doppler (DD) measurement mode, (b) Continuous Waveform (CW) measurement mode and (c) Time of Flight (TOF) measurement mode. The DD uses pulse compression sounding waveform to characterise the propagation path in terms of multipath delay, Doppler shift and Doppler spread measuring the channel scattering function. For instance, the waveforms used in Reference [35], which is one of the first DAMSON work published, is a 13 chips Barker sequence modulated at 2400 bps onto a bi-phase PSK carrier. Each code has a length of 5 ms, and is repeated 128 times every 15 ms providing and integration time of 1.92 s and a frequency resolution of 0.52 Hz. The range of the frequency Doppler is ±33 Hz and the processing gain due to the integration is <32 dB. However, the particular waveform in use and their characteristics can be configurable (an example of features and configuration parameters can be seen in Table 1), as well as a sample of its output in Figure 6. The CW measurement mode is a very sensitive detector because this mode uses a single frequency with a long integration time and low receiver bandwidth. It can be used for noise measurement as pre-scanning system. It also allows the receiver to automatically adjust its gain, whenever necessary, and determine large frequency shifts. Finally, the TOF measurement mode is similar to DD measurement mode with lower pulse repetition frequency. Consequently, the TOF is only able to measure the time of flight and high values of multipath delays due to it shorter integration time [35]. The DAMSON system is described in more detail in References [34,35].

#### 3.3.5. Results and Conclusions

Initial results measuring multipath, Doppler spread and Doppler shift with the whole system were presented in References [35,37]. The work presented in Reference [38] justifies the necessity of using an Automatic Radio Control System (ARCS) to select the best waveform to obtain a high data rate with an acceptable bit error rate, this analysis was done illustrating the range of Doppler shifts and spreads on a adverse high latitude HF channel.

The results for Doppler spread and Doppler shift during the campaign between 16 and 22 of October in 1995 is presented in Reference [39], where a disturbance takes place whereby the geomagnetic Q index reached values of 7 for several hour. This work demonstrated that the values of Doppler spread and shift are positively correlated with the level of disturbance. Multipath spread, Doppler spread and SNR were measured during the Spring, Summer and Autumn of 1995 for 4 high latitude paths in Reference [36]. Finally, these data were related to the performance of an HF data modem to estimate the availability on these paths. Doppler shift was also measured under both quiet and disturbed geomagnetic conditions in Reference [40]. In this work, longer multipath delays were observed during midday at frequencies above the predicted MUF, and higher Doppler spreads were observed during disturbed conditions, when the reflection point is located within the auroral oval. In Reference [41], the channel conditions measurements were used to evaluate the performance of two 75 bps (compliant with STANAG 4415 and STANAG 4285) and a 2400 bps waveforms, where the two 75 bps waveforms exhibited a 60–75% availability higher than the 2400 bps waveform, because the later was degraded during a geomagnetic disturbance.

The work presented in Reference [42] showed how the irregularities of the ionosphere at polar cap paths latitudes produce different angles of arrival of the transmitted signal at the receiver side, and how the movement of the reflection points produces both Doppler shift and Doppler spread. An additional high latitude paths were analysed in Reference [43], extending the campaign measurement time. The results of this new work concluded that this high latitude path presents less azimuth standard deviations than in polar cap paths, which means that the roughness of the ionosphere is less abrupt. A deep analysis was presented in Reference [44], where the direction of arrival of independent signal components were related to their precise Doppler frequency and with the ionospheric irregularity drift made by the CUTLASS (geophysical instrument) radars observation. The gross deviation of the displacement of the received signal from the great circle path is due to electron density depletion and ionospheric tilts within mid latitude through sub-auroral latitudes, whereas in the polar cap they are associated to the presence of patches and arcs of electron density. A review of the state of the art in this topic, quantifying and identifying the geophysical conditions that leads to this undesirable effect, is conducted in Reference [45]. In References [46,47], a spatial filtering has been implemented using simple arrays of antenna to reduce the level of Doppler spread, hence there is an improvement of up to 45% in the availability.

Channel simulation tools basically used the Watterson channel model, which is appropriate for mid latitudes paths. However, high latitudes paths are more complex than mid latitudes, more concretely when they have to take into account the directional effects. To tackle this issue in Reference [48], a campaign measurement took place between March 2004 and May 2005 over two paths: (a) from Svalbard to Kiruna, Sweden, and (b) from Kirkenes, Norway, to Kiruna. Finally, a channel model that incorporates this directional effects has been developed defining 7 different cases, which represents the complex range of measurements observed.

Finally, the channel characterisations obtained with the DAMSON project were very useful to define the requirements of the NATO robust HF waveforms: (a) STANAG 4415, which is also included in the (b) US Mil-Std 188-110A/B/C standards [49,50] and (c) in NATO STANAG 4539 [51], and also supported the HF automatic radio control system standard (d) STANAG 4538.

### 3.4. WHISPER

Wideband HF Ionospheric Sounder for Propagation Environment Research (WHISPER) project implements a pulse-compression HF ionospheric sounder using wide bandwidth (i.e., up to 80 kHz) to demonstrate the feasibility and benefits of wideband HF communications [28]. It transmits low power coded signals between remote sites of an oblique mid-latitude ionospheric link. It is a project mainly sponsored by UK Ministry of Defence with the collaboration of the Communications Research Centre in Ottawa, Ontario, Canada, under an UK-Canadian agreement on defence science and technology.

#### 3.4.1. Scope and Objectives

The goal of this project was to repetitively transmit pulse signals to compute the impulse response of the HF channel in wide bandwidths and study some features, such as signal time of flight, number of propagation modes, time dispersion, frequency dispersion and Doppler shift, fading statistics, absolute signal power, noise and interference. The point was to find out those channel characteristics that might impair wide-band transmission and then design proper signals characteristics to compensate most of them. A particular purpose was to investigate whether the Watterson [52] uncorrelated Rayleigh fading model, used to represent narowband channels (≤12 kHz) was applicable to wideband channels.

The main motivation of this project is to increase the HF link throughput to support user application growths. Previous research effort focused on increasing throughput on narrow-band HF channels (e.g, Reference [53]), but few studies ([54]) investigated using wider bandwidths. However, there was no consensus on which channel model was able to describe both the large channel features (e.g., Doppler shift and spread and multimode propagation) and the fine properties of these channels such as fading statistics (inter and intra mode). Therefore they proposed increasing the data rate of the HF channel not by increasing spectral efficiency in a standard 3 kHz bandwidth but using a wider bandwidth in order to use several simultaneous standard channels.

#### 3.4.2. System Deployment

Wideband sounding experiments were conducted over a 170 km path, East-West, from Malvern to Cobbet Hill radio station in UK during the spring of 2001. The transmitter at Malvern used a 100 W power amplifier which fed a droopy loaded dipole. The sounder receiver was located at Cobbet Hill, which is an electrically quiet radio station, and was fed from a wideband (2–8 MHz) dipole antenna.

Initial sounding experiments gathered wideband records of approximately 140 s duration every five minutes during day and night. PN-1023 sequences at a rate of 61.4 kchips/s were used to measure 8192 complex channel impulse responses. To decide which carrier frequency was used for each experiment a chirp sounder was located at the transmission site. Prior to each wideband transmission a 10 W linear chirp sounding from 2 to 16 MHz was performed. Moreover, this chirp signal was also used to generate ionograms and compute absolute time-of-flight due to the fact that both end links were synchronized to GPS time.

#### 3.4.3. Measurement Setup

WHISPER sounder is mainly composed of a wideband direct sampling HF transceiver, which is a software defined radio capable of digitize the whole HF band and downconvert to basedand and demodulate the channel of interest. It is designed as a digital HF radio modem on which an HF channel sounder can be implemented. The flexibility of this platform is based on the fact that its functionality is defined through the download of application software and Programmable Logic Device (PLD) configuration. The main parts and features of this software radio platform are the following:

- A PCI interface allow the transceiver to be installed on a host PC.
- A digital Transceiver Bus Arbitration based on a CPLD from Altera. It manages the interface to the local bus side of the PCI interface device, manages the bus interface to the DSP sub-system and allows software configuration of a processing FPGA.
- A Digital Signal Processing (DSP) sub-system. It is mainly composed of a dual Super Harvard Architecture (SHARC) processor (ADSP-21060) from Analog Devices module with a 2 Mbyte × 48 bit wide shared SRAM. This module undertakes all tasks related to the digital transceiver application and benefits from specific capabilities of SHARC processors: internal architecture able to process 80 million floating operation points per second (MFLOPS), high speed link port interfaces, high speed serial ports and optimised multi-processor cluster computing.
- A 100k gates FPGA is used to perform high speed or time critical processing in hardware and also allows data path to be configured as required for any particular application.
- 4-channel digital HF receiver and 4 channel digital HF transmitter exciter.
- Digital interfaces including synchronous and asynchronous serial data interfaces

**Figure 7:**
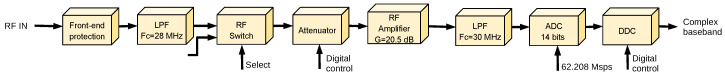
Block Diagram of WHISPER RF Front-end Direct Sampling HF Receiver

The transceiver includes a very important part, that is, a mixed analog and digital front-end which is responsible for the feasibility of a direct sampling HF receiver (see block diagram in Figure 7). Its main parts are:

- Front-End Protection and Antialiasing filter. It is a 13th order double stage elliptical low pass filter (LPF) with a 28 MHz cutoff frequency and with 120 dB selectivity well beyond 500 MHz.
- An RF switch allows the receiver to be switched between a number of sources.
- A digitally controlled attenuator (AT65-0223) and a fixed 20.5 dB gain RF amplifier(SNA-586) with a noise figure of 5.5 dB and an input −1 dB compression point of +19 dBm.
- A 5th order Chebychev harmonic LPF with a 30 MHz cutoff frequency reduces the level of internally generated out-of-band signals reaching the ADC.
- A 14b ADC from Analog Devices (AD6644) sampling at 62.208 Msps combined with a narrowband dither source reaches a Spurious Free Dynamic Range (SFDR) higher than 110 dB. It allows a Blocking Dynamic Range (BDR) of 114 dB inside a 3 kHz bandwidth.
- A Digital Down Converter (DDC), from Graychip GC4014 DDC, composed of a digital mixer and several decimation filters selects and downconverts the channel of interest.
  This front-end architecture allows to reach the following receiver characteristics: sensitivity of −113 dBm, input 3rd order interception point of +19 dBm, input 2nd order interception point of +27 dBm, blocking dynamic range within a 3 kHz bandwidth of +110 dBm and a noise figure of +17 dB.

#### 3.4.4. Measurement Techniques: Waveforms

The wideband sounder of WHISPER project used maximal length pseudo noise sequences (M-sequences) that were bi-phase shift keyed (BPSK) modulated. M sequences have good properties of auto-correlation and cross-correlation ([55]). Due to the requirements of bandwidth and delay time ranges they use M-sequences of length 511 and 1023 chips. They chose BPSK modulation since it is very simple to implement at baseband, it has constant amplitude and hence maximises the transmitted signal power and provides maximum distance between symbols, which is a very interesting characteristic in hostile HF channels. Table 2 summarizes the relevant characteristics of a number of waveform signals used as sounding signals with different bandwidth (chip-rate is ∼80% of the sounding bandwidth), including a narrowband variant compatible with those used in DAMSON project.

#### 3.4.5. Results and Conclusions

The outcomes of this project are given as three different study cases at three different frequencies—3.9 MHz, 5.7 MHz and 6.7 MHz [56]. They compute the Scattering function, the delay and Doppler power profile over 140 seconds of wideband PN received signals. By means of a chirp signal they can compute the state of the ionosphere simultaneously to the transmission of wideband PN signals. The goal is to compare both outcomes and better understand the behavior of the ionosphere.

##### Study Case 1: 3.9 MHz

In this case study gathered on 10th April 07:38 UTC they detected 4 propagation modes that, according the chirp ionogram, can be interpreted as 1E, 1F2, 2F2 and 3F2 modes. The power delay profile shows a delay spread around 4 ms and a Doppler spread of ±0.5 Hz. It is an example of a static channel that almost does not change throughout the 140 seconds of the experiment.

##### Study Case 2: 5.7 MHz

The second case study gathered on 9th April 20:31 UTC shows a more rapidly changing channel within the measure timescale than study case 1. The oblique ionogram shows multiple F-layer returns which corresponds with a series of five multipath modes discernable in the scattering function. The power delay profile shows a delay spread around 10 ms and a Doppler spread of of ±5 Hz.

##### Study Case 3: 6.7 MHz

The third case study gathered on 10th April 08:10 UTC shows once again an almost static channel throughout the 140 s of transmission. The scattering function shows a delay spread of 6 ms and a Doppler spread of ±2 Hz.

As a final conclusion, they present the WHISPER project as a feasible tool to sound the ionosphere in a wide bandwidth (i.e., wider than the standard 3 kHz), which is corroborated by a chirp sounder that simultaneously collect data on the same link. The results confirm the ability of the ionospheric sounder to measure the scattering function with enough multipath and Doppler resolution to measure each path amplitude and phase. This resolution is sufficient to identify mid-latitude multipath conditions which are likely to have an adverse effect on the majority of HF data modems.

### 3.5. Antarctica Project

The continent and the seas around the Antarctica are a natural laboratory of great interest for the scientific community. Several sensors are deployed to perform experiments for biology, geology and physics purposes with the aim to maintain an historic series or to carry out experiments that cannot be reproduced anywhere else on the Earth. The information obtained from these remote sensors should collected manually by the research community or be transmitted through satellite when the radio link is available. La Salle (http://www.salleurl.edu/en/research), together with Observatori de l’Ebre (OE) (http://obsebre.es/en/research), both part of the Universitat Ramon Llull (URL) (http://www.url.edu/en/research-and-innovation), have been partners of a research project on the Antarctica for remote sensing and sky wave digital communication modem design for more than ten years [57].

#### 3.5.1. Scope and Objectives

The Spanish Antarctic Station Juan Carlos I, on Livingston island, is only inhabited during the Austral summer, but remote sensors are measuring throughout the entire year. Much of this information must be processed in almost real time, so it cannot wait until the following Antarctic campaign. This information is transmitted to the OE in Spain through a satellite link. Currently, only part of the recorded geomagnetic data from just one of two variometers in operation can be sent to the OE by satellite link and made available using the facilities provided by the INTErnational Real-time MAGnetic observatory NETwork (INTERMAGNET).

The main objective of the Antarctica project was to deploy a trans-equatorial long-haul HF link, considered as an alternative to satellite or a complementary backup system in case of satellite link failure, to send data from several sensors in Antarctica to Spain. To fulfill this purpose, other major objectives have been derived: (a) obtain an historic series of data of the ionospheric channel for almost a solar cycle to characterise and understand this ionospheric channel and (b) define and evaluate HF data modem waveforms: this information will be used to specify both a robust and a high throughput modulation scheme depending on the status of the ionospheric channel. For more information about the motivation and the development of the project the reader is referred to Reference [57].

#### 3.5.2. System Deployment

The project was sounding the channel from 2003 to 2014, which is almost a complete solar cycle. Additional information about the duration of the last five campaigns can be found in Table 3.

**Figure 8:**
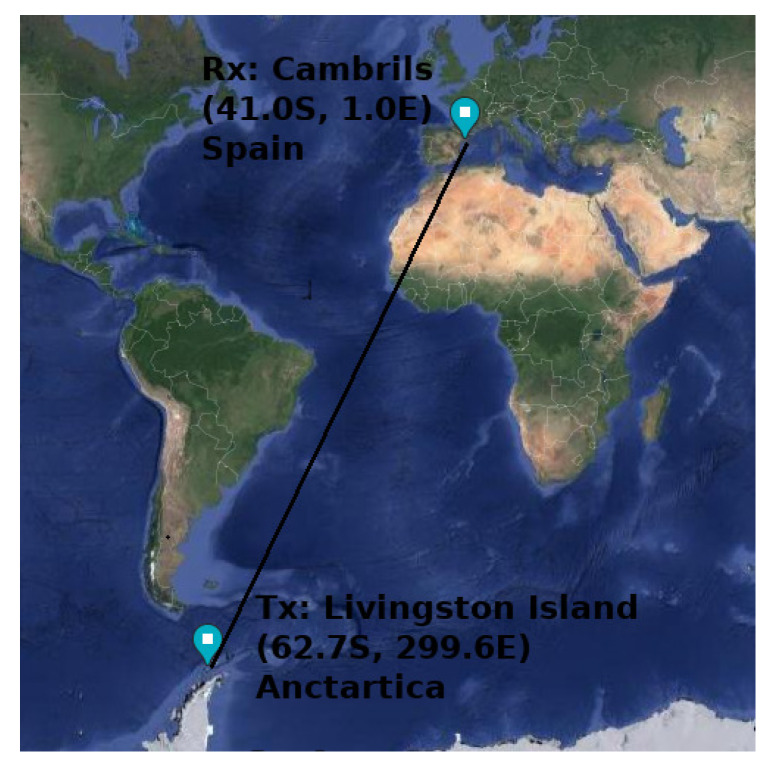
Geographical link characteristics. The transmitter is located in the Spanish Antarctic Station on Livingston Island, and the receiver is placed in Cambrils, Spain.

This system was deployed with one transmitter in Livingston island, Antarctica (62.7 ${}^{\circ }$S, 299.6 ${}^{\circ }$E), and one receiver with three different antennas in Cambrils, Spain (41.0 ${}^{\circ }$N, 1.0 ${}^{\circ }$E). The resulting ionospheric radio link is a trans-equatorial path, which is 12,760 km long. Figure 8 shows a map with geographical link characteristics.

**Figure 9:**
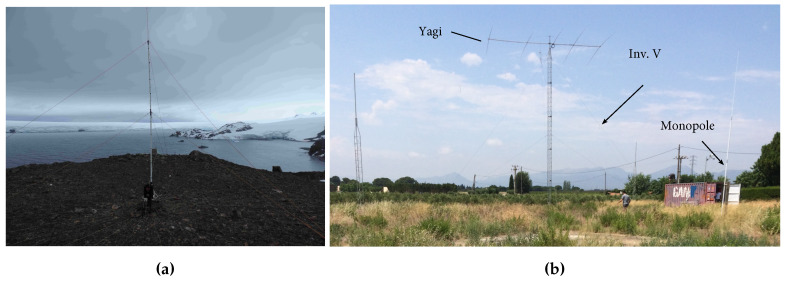
(**a**) The single transmitter antenna in Antarctica and (**b**) the three antennas of the receiver in Cambrils.

**Figure 10:**
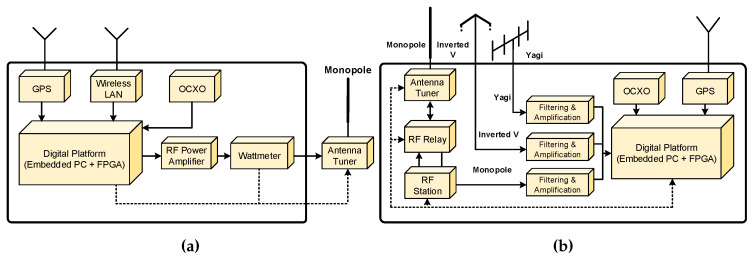
(**a**) Complete block diagram of the Antarctica project transmitter and (**b**) Complete block diagram of the Antarctica project receiver.

The transmitter was placed at the top of a hill close to the Station (see Figure 10a for a block diagram and Figure 9a for a picture of the location). The receiver was placed in Cambrils, a village located 100 km south of Barcelona, in a quiet electromagnetic environment. A block diagram of the receiver is shown in Figure 10b and a photograph is shown in Figure 9b.

#### 3.5.3. Measurement Setup

The Antarctic sounder is composed of a custom system that generates and processes the signals at transmitter and receiver, respectively [58,59]. The use of commercial components was discarded since the transmission was designed to use a bandwidth exceeding 3 kHz. The research group implemented a custom system to fit the strictest requirements of the channel. The solution was based on a software defined radio platform, whose last update was on the 2009/2010 campaign.

The main parts and features of this platform are the following:

- An embedded PC is used to control the entire system and storage data.
- A Virtex-4 XtremeDSP development platform from Xilinx, with three FPGAs that conduct all the signal processing operations needed, including the up and down conversion of the signal to be transmitted and received. This card is provided with 2xADC and 2xDAC of 14-bits. This systems is plugged to the embedded PC through PCI bus.
- A GPS unit was installed with the PPS signal, to obtain a time synchronization with an accuracy of up to 1 μs, since the absolute propagation time of the wave is a possible measurement in the receiver.
- A 100 MHz Oven Controlled Crystal Oscillator (OCXO) was installed in both the transmitter and the receiver side. The frequency stability in temperature of this oscillator is ±0.1 ppb.

The transmitter has also a Bonn HF power amplifier configured at 200 W of output power that feeds the antenna subsystem. The antenna subsystem is composed of a monopole of 7.5 m, several radials to improve the ground plane and an impedance tuner [26]. The signal to be transmitted is directly synthesized in the FPGA card by the DAC. The transmitter diagram block is depicted in Figure 10a.

The receiver is composed of three different antennas [58], a monopole, an inverted V and a Yagi with a resonance frequency of 14 MHz, in order to increase the information of the channel in terms of sounding. However, the monopole and the inverted V are not wideband antennas and a workaround has been implemented for each one. The inverted V requires a balun to flat the frequency response and an antenna tuner adapts the input impedance of the monopole to the input impedance of the receiver. The FPGA card samples the filtered signal provided by the antennas in their ADCs. Two software define radio platforms are required to process the data of the 3 antennas, since every system only contains 2 ADCs. The transmitter diagram block is depicted in Figure 10b.

The system is described in detail in Reference [58].

#### 3.5.4. Measurement Techniques: Waveforms Used

The Antarctic project performed the characterisation of the ionospheric channel with narrowband sounding to measure the SNR and the availability throughout a campaign and wideband sounding to measure the Doppler spread and the multipath delay spread through the scattering function. The soundings were performed during 24 h a day for a frequency carrier ranging from 2 to 30 MHz with steps of 500 kHz which corresponds to 57 sub-sessions. The frequency sampling of the band base signal is 100 ksps.

**Figure 11:**
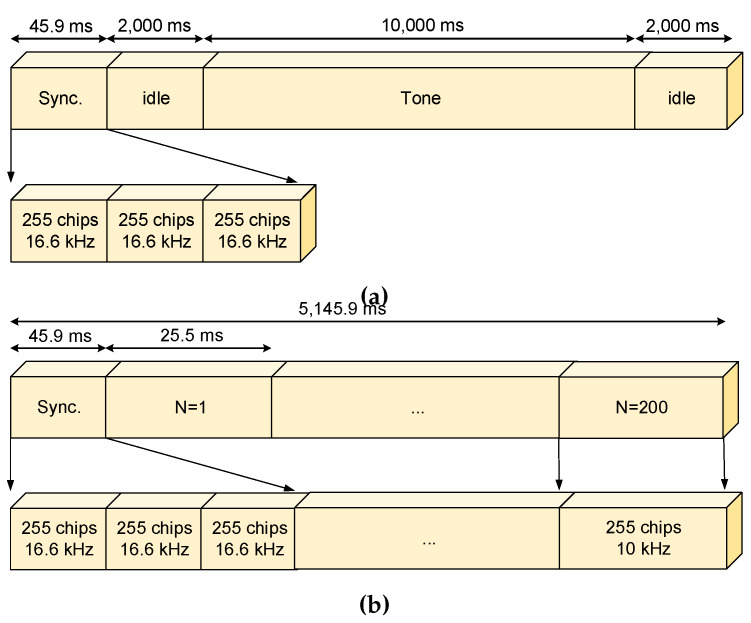
(**a**) Narrowband frame definition and (**b**) wideband frame definition.

The narrowband sounding frame (see Figure 11a) starts with a synchronization word, three PN m-sequence of 255 chips, then 2 s of silence followed by a transmission of a 10 s narrowband carrier and followed by 2 s of an idle slot again, as shown in Figure 11a. The idle periods are used in the receiver to measure the SNR and availability factor [26].

**Figure 12:**
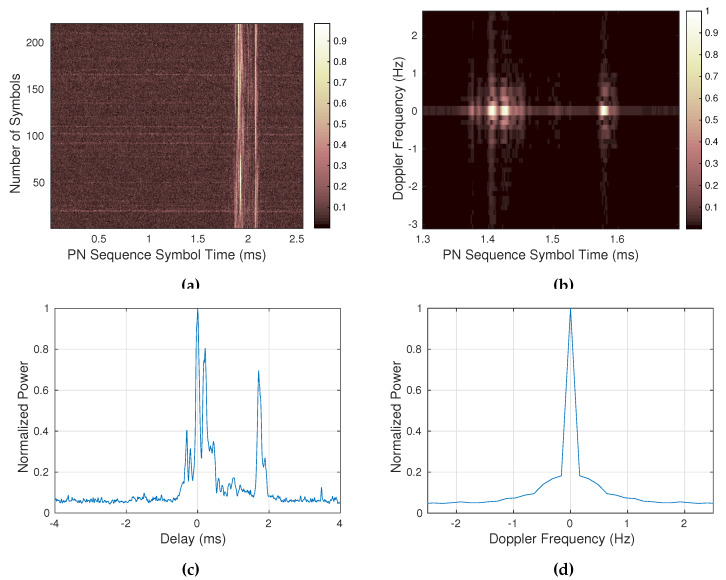
Wideband channel response for February 1st 2014 at 07 UTC with 13 MHz of carrier frequency: (**a**) normalized channel response *h*[*n*, *τ*], (**b**) normalized scattering function *R_s_*[*τ*, *v*], (**c**) multipath power profile (in ms), (**d**) Doppler power profile (in Hz).

The wideband sounding frame (see Figure 11) starts with a synchronization word, three PN m-sequences of 255 chips [32]. 5100 ms of wideband data is transmitted in this use case, using a PN m-sequence of 255 chips, obtaining a total of 200 consecutive sequences with a bandwidth of 10 kHz, from which the system derives the channel response matrix (see Figure 12a) and afterwards the scattering function (see Figure 12b). From these two functions the multipath delay spread (see Figure 12c) and the Doppler spread (see Figure 12d) of the channel will be calculated. Other characteristics, such as the SNR and even the number of paths could also be computed. The characteristics of the wideband sounding are summarized in Table 4.

#### 3.5.5. Results and Conclusions

This section highlights the main results obtained from the tests performed regarding the sounding of this long-haul trans-equatorial link between Antarctica and Spain. This information was essential for designing the appropriate physical layer in such a hostile channel.

The campaign 2005/2006 is presented in Reference [60], where 22 consecutive days were sounding from January to February 2006. The frequencies tested were in the range from 4 to 18 MHz, and bandwidths up to 16.5 kHz. This work present the propagation losses, delay and Doppler spreads and signal to noise and interference ratio.

The results of the campaign 2006/2007 is presented in Reference [61], corresponding to the soundings performed during 60 days from the 6th December 2006 to the 5th February 2007. The measurements include availability, multipath and Doppler spread and SNR. These measurements exhibited different results for daytime and nighttime. We can derive from this work that the maximum delay and the Doppler spread window values are [−3.5,3.5] (ms) and [−2.5,2.5] (Hz), respectively.

The experiments conducted in the 2013/2014 campaign is presented in Reference [26]. These soundings were carried out for 25 days, between 25 January and 18 February. In this work, the authors measured SNR, availability, multipath delay spread and Doppler spread.

In Reference [62], the frequency having the largest availability at a given time (FLA) measured in the Antarctica to Spain radio link has been correlated with the measurements of MUF(3000) of the vertical ionosonde (VIS) deployed along this radio link. The aim of this study is being able to estimate the best frequency of operation using the VIS information. This work has been extended in Reference [63] with more VIS and more campaigns (2009/2010, 2010/2011 and 2011/2012).

A polarisation diversity work was conducted in Reference [64], where both a monopole and a dipole (inverted-V) at reception were the antennas used in this campaign. These receiving antennas are orthogonal polarized to check whether wave polarization rotation causes differences between simultaneous signals. Polarisation diversity could be used to increase SNR and performance through smart combination of receiving signals, due to the low cross-correlation factor between them.

Throughout the sounding campaigns four different zones can be distinguished analyzing channel availability: day, night, dawn and dusk. These four time zones differ in the frequency that exhibits better performance in terms of availability, which is the condition for the change of zone. These differences are summarized in Table 5.

The best reflective frequency during the nighttime are the lowest ones. And the best during daytime are the highest ones. During the day, from 10 to 17 UTC, 22-23 MHz is the optimum frequency in terms of availability, and during the night, from 21 to 06 UTC, two bands nearby 9 MHz and 12 MHz report the largest availability. As sunrise approaches, the Frequency of Optimum Transmission (FOT) increases from 9 to 23 MHz and the FOT decreases from 23 to 12 MHz in the sunset period.

**Figure 13:**
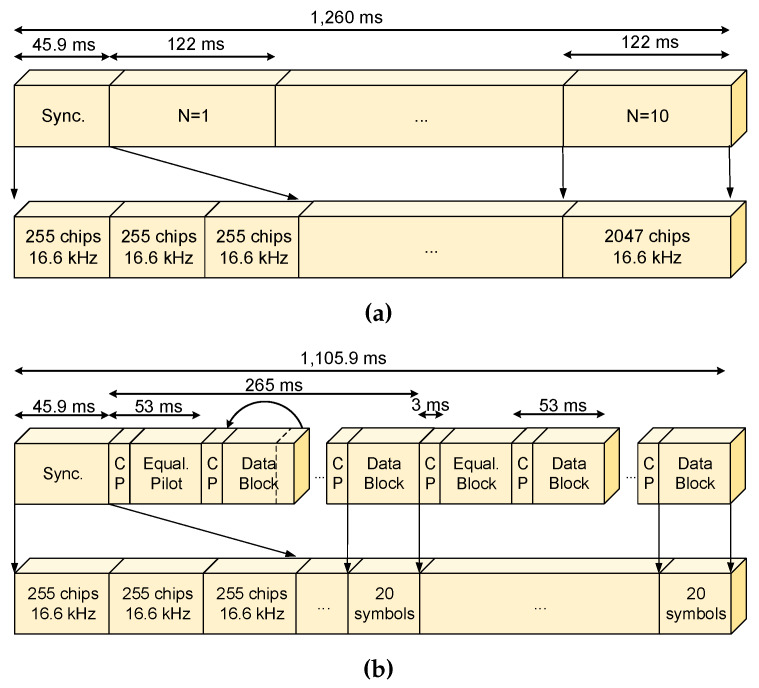
(**a**) High robustness data frame definition and (**b**) high throughput data frame definition.

Finally, two different frame structures were defined to adapt the system to the ionosphere conditions [57]: (i) a frame aiming to high robustness data transmission (Figure 13a and (ii) a frame for high throughput data transmission (Figure 13b). The difference between these two last options is the data throughput and the chosen modulation, which has been designed based on the results of the soundings during several Antarctic campaigns [65].

## 4. Conclusions

The HF communications allow transmitting signals beyond line-of-sight propagating them through the ionosphere. The ionospheric effects lead to the degradation of the signal suffering attenuation, time dispersion and frequency dispersion (Doppler spread and Doppler shift). HF sounding of the ionosphere has been of great interest for research community to obtain knowledge about the behavior of the plasma that comprises the different reflected layer of the channel and its influence in the signal impairments.

HF ionospheric communications systems present mechanisms that enable communication in a large area without the need of a network infrastructure, satellites or repeaters. This possibility of independence of local infrastructure is absolutely crucial for disaster relief communications, when the infrastructure is usually absolutely destroyed by a large scale natural disaster, or in remote regions where this infrastructure is lacking, as the Antarctica.

This research work has conducted a review of an ITU-R on recommendation on HF field-strength measurement campaigns. Afterwards, vertical sounding is addressed with a world wide network of ionosondes (GIRO), and finally, three oblique sounding projects have been reviewed for high latitude (DAMSON), mid latitude (WHISPER), and trans-equatorial of 12,760 km latitude (Antarctica).

The review shows that during the last decades a huge progress has been made in the design of ionospheric sounding systems to adjust the construction of HF inospheric communication systems. Even so, in the design of any new communication system, the previously detailed techniques should be applied to check the reaction of the channel under study to the different modes of propagation and communication.

## Figures and Tables

**Table 1:** Characteristics of different DAMSON sounder waveforms (3 kHz bandwidth) [34].

Delay	Number	Doppler	Doppler	Integration
Range	of Frames	Range	Resolution	Period
(ms)		(Hz)	(Hz)	(s)
5	64	±100	3.125	0.32
5	128	±100	1.563	0.64
5	256	±100	0.782	1.28
5	512	±100	0.391	2.56
10	64	±50	1.563	0.64
10	128	±50	0.782	1.28
10	256	±50	0.391	2.56
10	512	±50	0.195	5.12
15	64	±33	1.042	0.96
15	128	±33	0.521	1.92
15	256	±33	0.260	3.84

**Table 2:** Characteristics of different WHISPER sounder waveforms

Waveform	Chip-Rate	Delay	Multipath	Doppler	No. of	Doppler	Measure	Processing
BPSK	(kchip/s)	(ms)	Res. (μs)	(Hz)	CIRs	Res. (Hz)	Time (s)	Gain (dB)
PN-1023	81	12.6	∼10	±40	8192	0.01	103	70
PN-1023	61.4	16.6	∼15	±30	8192	0.008	136	70
PN-511	40.8	12.5	∼35	±40	8192	0.01	102	67
Barker-13	2.4	12.5	∼600	±40	128	0.06	1.6	32

**Table 3:** Start date, end date, and number of days per survey for the last five studied campaigns.

Survey	Start	End	#
	Date	Date	Days
2009/2010	01/01/2010	02/02/2010	33
2010/2011	22/01/2011	01/03/2011	39
2011/2012	13/02/2012	25/02/2012	13
2012/2013	05/01/2013	24/02/2013	51
2013/2014	24/01/2014	18/02/2014	26

**Table 4:** Characteristics of the wideband sounding waveform of the Antarctica project

Waveform	Chip-rate	Delay	Multipath	Doppler	No. of	Doppler	Measure	Processing
BPSK	(kchip/s)	(ms)	Res. (μs)	(Hz)	CIRs	Res. (Hz)	Time (s)	Gain (dB)
255 m-sequence	10	25.5	∼100	±19.61	200	0.196	5.1	47

**Table 5:** Summary of the results for the narrowband analysis in terms of availability by time zones

Zone	Interval (UTC)	Usable Frequencies [MHz]
Dawn	07–09	Increases from 6–15 to 20–30
Day	10–17	20–30
Dusk	18–20	Falls from 20–30 to 10–20
Night	21–06	06–15

## Abbreviations

The following abbreviations are used in this manuscript:

ADC	Analog to Digital Converter
AGC	Automatic Gain Control
ARCS	Automatic Radio Control System
ASAPS	Advanced Stand Alone Prediction System
BDR	Blocking Dynamic Range
BLOS	Beyond line-of-sight
BPSK	Bi-Phase Shift Keyed
CW	Continuous Waveform
CRC	Communications Research Centre
DAC	Digital to Analog Converter
DD	Delay Doppler
DDC	Digital Down Converter
DERA	Defence Evaluation Research Agency
DSP	Digital Signal Processing
EO	Ebro Observatory
FFI	Forsvarets Forskningsinstitutt
FOA	Försvarets Forskningsanstalt
FOT	Frequency of Optimum Transmission
FPGA	Field Programmable Gate Array
GAMBIT	Global Assimilative Model of Bottomside Ionosphere Timelines
GPS	Global Positioning System
HF	High Frequency
ICEPAC	Ionospheric Communications Enhanced Profile Analysis and Circuit Prediction Program
INTERMAGNET	INTErnational Real-time MAGnetic observatory NETwork
IONCAP	Ionospheric Communications Analysis and Prediction Program
INAG	Ionosonde Network Advisory Group
ITU-R	The Radiocommunications section of International Telecommunications Union
IRE	Institute of Electronic Engineers
LGDC	Lowell GIRO Data Center
LOS	Line-of-Sight
MFLOPS	Million Floating Operation Points Per Second
NVIS	Near Vertical Incidence Sounding
OCXO	Oven Controlled Crystal Oscillator
PC	Personal Computer
PLD	Programmable Logic Device
PPS	Pulse per Second
SFDR	Spurious Free Dynamic Range
SHARC	Super Harvard Architecture
SNR	Signal to Noise Ratio
SSB	Single-Sideband
TID	Traveling Ionospheric Disturbance
TOF	Time of Flight
VOACAP	Voice of America Coverage Analysis Program
